# Supplementary material for: Consecutive prediction of adverse maternal outcomes of preeclampsia, using the PIERS-ML and fullPIERS models: A multicountry prospective observational study
Source: PLoS Med. 2025 Feb 4;22(2):e1004509. doi: 10.1371/journal.pmed.1004509 (PMC11793762; doi:10.1371/journal.pmed.1004509)
Supplement: S1 Table — (DOCX) [file pmed.1004509.s002.docx]

| **Outcome/Variable** | **Definition** |
| --- | --- |
| Ethnicity |  |
| White | Origins in Europe, Middle East, North Africa [Arabic origins], Western Russia [including Afghanistan and South Russia] and Hispanics of European origin |
| Asian | Origins in the Indian sub-continent [e.g., India, Pakistan, Bangladesh, and Sri Lanka], or in the Far East and Southeast Asia [e.g., China, Japan, Korea, Philippines, Thailand, Eastern Russia] |
| Black | Origins in any of the original peoples of Africa |
| Other | Including mixed ancestry |
| Mortality | Maternal death occurring within six weeks of pregnancy or if later, attributable to complications of preeclampsia |
| Hepatic dysfunction | International normalized ratio (INR) >1.2 in the absence if disseminated intravascular coagulation (DIC) or treatment of warfarin (DIC is defined as having both: abnormal bleeding and consumptive coagulopathy [i.e., low platelets, abnormal peripheral blood film, or one or more of the following: increased INR, increased prothrombin time (PTT), low fibrinogen, of increased fibrin degradation products that are outside normal non-pregnancy ranges]) |
| Hepatic hematoma or rupture | Blood collection under the hepatic capsule as confirmed by ultrasound or laparotomy |
| Glasgow coma score (GCS) <13 | Based on GCS scoring system: Teasdale G, Jennet B. Assessment of coma and impaired consciousness: a practical scale. *Lancet* 1974; **2**:81-83 |
| Stroke | Acute neurological event with deficits lasting longer than 48 hours |
| Cortical blindness | Loss of visual acuity in the presence of intact papillary response to light |
| Reversible Ischemic Neurologic Deficit (RIND) | Cerebral ischemia lasting longer than 24 hrs but less than 48 hours revealed through clinical examination |
| Retinal detachment | Separation of the inner layers of the retina from the underlying retinal pigment epithelium (RPE, choroid) and is diagnosed by ophthalmological exam |
| Acute renal insufficiency | For women with no underlying renal disease, defined as serum creatinine >150 μM |
| Acute renal failure | For women with an underlying history of renal disease, defined as serum creatinine >200 μM |
| Dialysis | Including haemodialysis and peritoneal dialysis |
| Postpartum hemorrhage (PPH) requiring transfusion or hysterectomy | Occurrence of PPH that required transfusion or hysterectomy |
| Placental abruption | Any occurrence of abruption diagnosed clinically or based on placental pathology report |
| Platelet count < 50,000 x 10^9^/L without blood transfusion | Measurement of platelet count recorded as less than 50,000 x 10^9^/L without patient receiving a blood transfusion |
| Transfusion of blood products | Includes transfusion of any units of blood products: fresh frozen plasma (FFP), platelets, red blood cells (RBCs), cryoprecipitate (cryo) or whole blood |
| Positive inotropic support | The use of vasopressors to maintain a systolic blood pressure >90 mmHg or mean arterial pressure >70 mmHg |
| Myocardial ischemia/infarction | Electrocardiogram (ECG) changes (ST segment elevation or depression) without enzyme changes AND/OR any one of the following: 1) Development of new pathologic Q waves on serial ECGs. The patient may or may not remember previous symptoms. Biochemical markers of myocardial necrosis may have normalized, depending on the length of time that has passed since the infarct developed. 2) Pathological findings of an acute, healed or healing MI 3) Typical rise and gradual fall (troponin) or more rapid rise and fall (CK-MB) of biochemical markers of myocardial necrosis with at least one of the following: a) ischemic symptoms; b) development of pathologic Q waves on the ECG; c) ECG changes indicative of ischemia (ST segment elevation or depression); or d) coronary artery intervention (e.g., coronary angioplasty) |
| Eclampsia | Any episode of seizure antepartum, intrapartum or before postpartum discharge as follow-up beyond discharge is not possible |
| Require >50% oxygen for greater than one hour | Oxygen given at greater than 50% concentration based on local criteria for longer than 1 hour |
| Intubation other than for Caesarean section | Intubation by endotracheal tube |
| Severe breathing difficulty | Suspected pulmonary oedema where x-ray confirmation unavailable may be diagnosed by presence of chest pain or dyspnea, crackles in the lungs and SaO_2_ <90% |
| Pulmonary oedema | Clinical diagnosis with x-ray confirmation or requirement of diuretic treatment and SaO_2_ <95% |
