# Supplementary material for: Consecutive prediction of adverse maternal outcomes of preeclampsia, using the PIERS-ML and fullPIERS models: A multicountry prospective observational study
Source: PLoS Med. 2025 Feb 4;22(2):e1004509. doi: 10.1371/journal.pmed.1004509 (PMC11793762; doi:10.1371/journal.pmed.1004509)
Supplement: S2 Table — #: Numbers in this column refer to the number of certain outcome events occurring at any time following admission in the pooled database used in this study. (DOCX) [file pmed.1004509.s003.docx]

| **Predictors** | | **Outcome component (N = 1083)#** |
| --- | --- | --- |
| **PIERS-ML model** [1] | **fullPIERS model** [2] |  |
| **Characteristics**:  gestational age on admission (weeks), maternal age at expected delivery date (years), height on admission (cm); weight on admission (kg), national maternal mortality ratio (per 100,000 live births), national *per capita* GDP ($USD)    **Biomarkers**:  Oxygen saturation (%), total leucocyte count; platelet count (10^9^/L), uric acid (mmol/L); serum creatinine (μmol/L), aspartate transaminase (U/L), alanine transaminase (U/L), mean platelet volume (fL), hematocrit (%), serum albumin (g/L), systolic blood pressure (mm Hg), diastolic blood pressure (mm Hg) | **Characteristics**:  gestational age on admission (weeks)  **Symptoms**:  chest pain or dyspnea  **Biomarkers**:  Creatinine (μmol/L), platelet count (10^9^/L), aspartate transaminase, oxygen saturation (%) | maternal death (2)  **Central nervous system**:  eclamptic seizure(s) (75); Glasgow coma score less than 13 (20); stroke or reversible ischemic neurological deficit (6); transient ischemic attack (1); cortical blindness (6); posterior reversible encephalopathy (5)  **Cardiorespiratory**:  positive inotropic support required (7); infusion of a third injectable antihypertensive (33); myocardial ischemia or infarction (6); oxygen saturation less than 90% (103); at least 50% fractional inspired oxygen for at least one hour (72); intubation other than for Caesarean birth (47); pulmonary oedema (96)  **Hematological**:  blood transfusion (460); platelet count less than 50x109 per L, without transfusion (111)  **Hepatic**:  Dysfunction (44); hematoma or rupture (0)  **Renal**:  acute renal insufficiency in women without chronic kidney disease (9); acute renal failure in women with chronic kidney disease (52); dialysis (11)  **Other**:  severe ascites (65); Bell’s palsy (6); placental abruption (129) |

Reference

1. Montgomery-Csoban T, Kavanagh K, Murray P, Robertson C, Barry SJE, Vivian Ukah U, et al. Machine learning-enabled maternal risk assessment for women with pre-eclampsia (the PIERS-ML model): a modelling study. Lancet Digit Health. 2024;6(4):e238-e50. doi: 10.1016/S2589-7500(23)00267-4. PubMed PMID: 38519152; PubMed Central PMCID: PMCPMC10983826.

2. von Dadelszen P, Payne B, Li J, Ansermino JM, Pipkin FB, Côté A-M, et al. Prediction of adverse maternal outcomes in pre-eclampsia: development and validation of the fullPIERS model. Lancet. 2011;377(9761):219-27. doi: 10.1016/s0140-6736(10)61351-7.
