# Supplementary material for: Consecutive prediction of adverse maternal outcomes of preeclampsia, using the PIERS-ML and fullPIERS models: A multicountry prospective observational study
Source: PLoS Med. 2025 Feb 4;22(2):e1004509. doi: 10.1371/journal.pmed.1004509 (PMC11793762; doi:10.1371/journal.pmed.1004509)
Supplement: S1 Text — (DOCX) [file pmed.1004509.s009.docx]

1. Precision recall curve

|  | Reality (+) | Reality (-) |
| --- | --- | --- |
| Prediction (+) | True positive (a) | False positive (b) |
| Prediction (-) | False negative (c) | True negative (d) |

Precision refers to the positive predictive value of a prediction model at a certain cut-off value of predicted probability (calculated as counts of true positive cases divided by the counts of predicted positive cases: a / (a + b)), and recall refers to the sensitivity (calculated as counts of true positive cases divided by the total actual positive cases: a / (a + c)) also at a certain cut-off of predicted probability.

Here is an example of precision recall curve (Precision recall curve of the fullPIERS model on day 0) which plots precision (y axis) against recall (x axis):


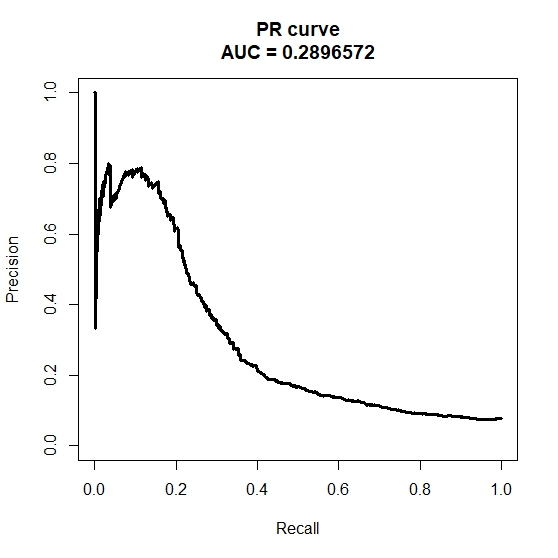


Area under the precision recall curve (AUC-PRC) refers to the total area between the precision recall curve and the x axis, similar to the concept of area under the receiver operating characteristic curve (AUC-ROC). Higher values of AUC-PRC indicate better model performance.

1. Decision curve analysis

Decision curve analysis (DCA) is employed to evaluate the clinical utility of prediction models.[1,2] The key metric that measures clinical utility in DCA is “net benefit”. The net benefit of a prediction model under study is compared to the situation where no prediction model is available (thus clinicians can only have the “treat all” or “treat none” policy). At a pre-defined cut-off value of the prediction model (termed as “threshold probability” in DCA ), if the model shows fairly higher net benefit compared to the “no model” situation, then we may think this model is clinically useful. DCA plots net benefit (y axis) against the whole range of plausible threshold probabilities (x axis) so we get a better picture of the clinical utility of the model under study.[2]

Then how do we calculate net benefit of a prediction model?

Let *pt* denote threshold probability, net benefit = $\frac{True positive count}{n}-\frac{False positive count}{n}*\frac{pt}{1-pt}$,[2] where n refers to the total number of subjects, “*True positive count*” refers to the number of subjects that are truly positive and are also predicted to be positive by the model at a certain *pt*, and “*False positive count*” refers to the number of subjects that are actually negative but are predicted to be positive by the model. The detailed reasoning of this formula can be found in the original article.[2]

How do we calculate net benefit of the “treat all” or “treat none” policy?

The formula for calculating the net benefit of the “treat all” or “treat none” policy is the same one shown above. In fact, we can think of the treat all or none policy as the simplest prediction model. Specifically for the “treat none” policy, the net benefit stays 0 as both the “*True positive count*” and “*False positive count*” are 0, thus we often mention the comparison between the model and the “treat all” policy.

In our study, four pre-defined threshold probabilities (calculated based on likelihood ratios), i.e. 0.6%, 3.1%, 18.8%, 45.6%, stratify patients into very low-risk, low-risk, moderate risk, high-risk and very high-risk groups.[3] If clinicians decide to initiate immediate delivery to, for example, all patients in the high- risk and very high-risk groups, the threshold probability would be 18.8%, then we can tell from the decision curve analysis plots if the net benefit of the model is higher than that of both the “treat all” policy and “treat none” policy (which is always 0) on each day, and the change of the relative advantage (if there is any) of the model over other policies over time.

1. Sankey diagram (more details of Fig 4)

Fig 4 shows a Sankey diagram. Each risk stratum (from very low risk to very high risk) has its own flow characterized by a unique color (e.g., dark blue color for the very low risk group, and orange color for the very high risk group) from day 0 to day 13. Each day forms a node in the flow. Apart from the risk strata, we also show whether patients had adverse outcome (denoted by “outcome”) or uncomplicated course (denoted by “delivery: no complication”) on the figure.

The width of the flow is proportional to the number of women it represents. Thus we can tell that on day 0, the moderate risk strata has the highest number of women, and the very high risk strata the lowest. From day 0 to day 1, we can see in the very high risk that: (1) almost half of the women group developed adverse outcomes (as almost half of the red color flows into “outcome”); (2) around 30% of the women stayed in the very high risk group (as around 30% of the red color flows into “very high”); (3) around 15% of the women switched to the high risk group; and around 5% of the women switched to the moderate risk group. We can read the results on other days and for other risk groups following the same way described above.

As time goes on, the dark band (“outcome”) gets wider as more adverse outcome events happened, and the white bar (“delivery: no complication”) gets longer as more deliveries happened. The positions of the band and the bar changed sometimes due to the lack of a risk group (e.g. on day 12 no women were predicted to be at very high risk).

References

1. Collins GS, Moons KGM, Dhiman P, Riley RD, Beam AL, Van Calster B, et al. TRIPOD+AI statement: updated guidance for reporting clinical prediction models that use regression or machine learning methods. BMJ. 2024;385:e078378. Epub 20240416. doi: 10.1136/bmj-2023-078378. PubMed PMID: 38626948; PubMed Central PMCID: PMCPMC11019967.

2. Vickers AJ, Elkin EB. Decision curve analysis: a novel method for evaluating prediction models. Med Decis Making. 2006;26(6):565-74. Epub 2006/11/14. doi: 10.1177/0272989X06295361. PubMed PMID: 17099194; PubMed Central PMCID: PMCPMC2577036.

3. Montgomery-Csoban T, Kavanagh K, Murray P, Robertson C, Barry SJE, Vivian Ukah U, et al. Machine learning-enabled maternal risk assessment for women with pre-eclampsia (the PIERS-ML model): a modelling study. Lancet Digit Health. 2024;6(4):e238-e50. doi: 10.1016/S2589-7500(23)00267-4. PubMed PMID: 38519152; PubMed Central PMCID: PMCPMC10983826.
