# Supplementary material for: Consecutive prediction of adverse maternal outcomes of preeclampsia, using the PIERS-ML and fullPIERS models: A multicountry prospective observational study
Source: PLoS Med. 2025 Feb 4;22(2):e1004509. doi: 10.1371/journal.pmed.1004509 (PMC11793762; doi:10.1371/journal.pmed.1004509)
Supplement: Attachment — Submitted filename: Response to reviewers.docx [file pmed.1004509.s010.docx]

**Comments from** **the editors**:

In line with the comments from the statistical reviewer (Reviewer 3), we would like to see external validation of the model using an independent dataset in a revised version of the manuscript. We would also like to see the outcomes of testing the models using follow-up data, as per Reviewer 3's comments.

Thank you very much for your valuable suggestions and comments. We apologize for any confusion and for not providing clearer content. In brief, this project was not designed to develop a new model, but rather to test the performance of serial use of the existing static PIERS models. It is worthy of stating that **both PIERS models used in this study have been externally validated previously**. More specifically, the fullPIERS model has undergone comprehensive external validation.[1-4] Until the PIERS-ML model was introduced this year, the fullPIERS model was the best in predicting the adverse maternal outcomes of pre-eclampsia.[5] The PIERS-ML model had a high value of area under the receiver operating characteristic curve (AUROC) on the external validation dataset: 0·80 [95% CI 0·76–0·84],[5] which shows very low risk of overfitting.

In this study, we aim to signal and quantify performance drift of these two models used for consecutive prediction on the **follow-up** **data** **from the largest dataset** in this field, because the National Institute of Health and Care Excellence (NICE) guideline in the United Kingdom suggests that the fullPIERS model be used iteratively for consecutive prediction for the same woman, when the model has never been designed and validated for such use.[6] Based on our findings, clinicians should interpret the risks obtained by serial use of the PIERS models (for want of better alternatives) with caution. Actually, we are working on dynamic prediction models that incorporate the trajectory of predictors (instead of merely one measurement) for estimating risks. Once completed, we can compare the performance of our dynamic prediction models to the results in this study (where static models are used for consecutive prediction) to quantify the added value of dynamic prediction models.

In the meantime, there seems to be a misunderstanding between the editors and Reviewer 3. As Reviewer 3 acknowledged, this study was simply an application of existing, validated models on follow-up data the models were not intended to be used for, which reflects the actual clinical use of the models. Reviewer 3 mentioned that we could develop (and validate) separate PIERS-ML models for consecutive/ongoing prediction with the follow-up data, which could be a solution to the problem of performance drift of the static models, and we also mentioned this approach in the section of “*Potential solutions*” under “Discussion” (see lines 373 to 375 in the clean copy): “*For consecutive prediction using serial measurements, existing prediction models can be updated or new models trained for this purpose. Potential approaches include recalibrating intercept or both intercept and joint effects of predictors*…”. However, in this approach we would still only use one measurement of predictors to develop models; as mentioned previously, we prioritize working on dynamic prediction models in which we hope to incorporate the added value of the trajectory of predictors, which also resembles repeated assessment in clinical practice.

**Comments from the** **Reviewer 1**:

Reviewer #1: This was a prospective observational study evaluating the performance of the PIERS-ML and fullPIERS models for consecutive prediction of adverse maternal outcomes in women with preeclampsia, beyond their initial 48-hour assessment window in large cohort (8,843) of pregnancies.
It demonstrates that  both models exhibited a decline in predictive accuracy over time and were most predictive at the first assessment point within 48 hours of presentation.
The study recommends that the models are only used for initial testing. They suggest that there is a need for a more dynamic model that accommodates changing measurements and the effects of therapy. This should lead to a change in guidance.
However, even though there is a performance drift, there is still some predictive value that can be used along with clinical assessments, especially at the extreme ends. So the models are not invalid but could be seen as a clinical support tool that could be improved by additional research using modern techniques.

Thank you very much for the insightful comment. Indeed, the models had useful performance characteristics beyond the initial day of assessment. We aimed to demonstrate that while these characteristics are still acceptable to some extent, the model performance reported on day of admission is not an accurate representation of model performance over time, and as this performance becomes unreliable, it would be more appropriate to create new models which can take the medical history since admission into account.

This is particularly true in resource limited settings were continuous real-time assessments may not be feasible. The PIERS models could still be useful.
Risk assessment is just that, assessing risk. the clinical care needs to be adapted by the patients’ wishes and the clinical developments.
While the study provides important insights into the limitations of the PIERS-ML and fullPIERS models for consecutive predictions, these models still hold value in clinical practice. A more balanced approach that focuses on improving the models and integrating them into a broader clinical framework, rather than limiting their use, would better serve the needs of patients and clinicians.
Until further new guidance is produced of a more dynamic model, it would be premature to recommend not to use the models beyond the first assessment.

We totally understand your point and fully agree with you. Thank you very much. We have revised our conclusions accordingly (see lines 395 to 401 in the clean copy): “*Based on our findings of performance drift (especially in terms of clinical utility) and the lack of better alternatives, we recommend using both the PIERS-ML and fullPIERS models for consecutive prediction of adverse maternal outcome in preeclampsia more cautiously as pregnancy progresses. Clinicians should interpret consecutive predictions with increasing uncertainty. To optimize maternal outcomes, future work may consider developing a dynamic approach, to account for individual trajectories so that predictions can be updated serially. The performance of these dynamic approaches can be compared with the results of this study to quantify their added value*.”

**Comments from Reviewer 2**:

Thank you for asking me to review this interesting manuscript which addresses the value of repeated application of a predictive model to detect adverse outcome in preeclampsia. The two tools evaluated included the (original) full PIERS model and the (recent) PIERS Machine Learning model. This latter model includes more predictor variables, and has been developed- and validated- in a multiethnic population, published in Lancet Digital Health this year. It outperforms the full PIERS model, in part (i) because of it adds baseline adjustment for national GDP and MMR to the model and (ii) it has removed symptoms, with associated subjectivity. The ML PIERS model was able to stratify maternal risk, with high LR and event rate within 48 hours accompanying those classified as high or very high risk, and very low event rates among those classified as lowest risk. Knowing the risk of severe adverse outcome in the next 48 hours can be used to inform care decisions such as transfer, admin of corticosteroids etc.

The question asked in this paper is whether the PIERS model (either full PIERS or ML PIERS) can be reapplied daily during the disease course to better identify women at high risk of adverse outcome beyond the initial triage. Perhaps unsurprisingly, the performance of this model decays over time post admission.
1. This may be, firstly, due to predictors indicating an outcome already present, eg oxygen saturation as a predictor- and oxygen saturation less than 90% as an outcome, which inflates 'predictive' power.  Do you have data on what proportion of patients this applied to?

Thank you for bringing up this important point, which we carefully considered during our modeling process. In brief, we included "oxygen saturation" as both a predictor and a component of the outcome because we were interested in predicting the development of adverse events based on information available at admission. To ensure the integrity of the predictions, we only used data from before the occurrence of the first adverse maternal outcome. In addition, previously, we determined that pulse oximetry is predictive of non-cardiopulmonary outcomes in women with pre-eclampsia.[7]

This approach ensured that while oxygen saturation was both a predictor and part of the outcome, no predictions were made on data where oxygen saturation had already fallen below 90. If a patient's oxygen saturation dropped below 90, that was identified as an outcome, and any subsequent data from that patient was excluded from the analysis. This methodology ensured that we were not predicting oxygen saturation <90 with an oxygen saturation value that was already <90.

2. The presence of some of these predictors will necessitate immediate delivery. This means the worst group- where the predictive model will perform best- is rapidly removed from the cohort. Although the cohort has been described elsewhere, some baseline demographics such as gestational age and whether patients were universally undergoing expectant management would help to interpret threshold for delivery.

Thank you very much for your insightful comments. We agree that patients who were identifiably at the highest risk on or soon after admission would be delivered quickly and removed from the data. We believe this strengthens our conclusion that the static model developed on day of admission should be used with greater caution beyond a day or two after admission, as the removal of the patients at the highest risk in clinical practice could create a cohort that is significantly different from the cohort the model was developed on.

We acknowledge that the models were developed and validated using real-world data, where careful maternity care providers responded to clear evidence of maternal or perinatal risk. This becomes clear in the PIERS-ML paper, in which the stillbirth rate was lower in the very-high risk stratum of women compared with the high risk stratum. Sites that contributed data to this combined cohort had a general policy of expectant management for women with preterm pre-eclampsia and, post-HYPITAT, a general policy of induction at term. However, no sites had hard and fast policies that overrode individual clinician decisions. We have clarified this in our manuscript (see lines 108-110 in the clean copy).

According to your suggestion, Table S1 is added that shows the baseline characteristics of the women. The gestational age of the women at admission ranges from 30.57 to 38.63 and the women were recruited between year 2003 and 2016 (see lines 93 – 94 in the clean copy).

3. For those remaining, intervention may also prevent the adverse outcome (e.g. controlling severe HT to reduce CNS sequelae). The impact of treatment/ intervention is not accounted for in the model, which will limit its applicability across time.

We fully understand your concern regarding the lack of direct consideration for the impact of treatment or intervention in the model which is indeed a frequently asked question. However, interventions (e.g., varying drugs at varying intervals and doses, fluids) are too complex to track, and, probably because of this, are not used in Acute Physiology and Chronic Health Evaluation (APACHE, for ICU) or Score for Neonatal Acute Physiology (SNAP, for neonates). Moreover, we would like to clarify that the severity and prognostication of a disease/syndrome are fundamentally assessed through biomarkers, such as blood pressure and platelet counts. The effects of any treatment or intervention are inherently reflected in these biomarker values. Consequently, while the model does not explicitly include intervention as a predictor, its impact is indirectly accounted for through the biomarker measurements.

Additionally, as interventions are often reflected by the magnitude and/or changes in biomarker values, and the biomarkers post-intervention already encapsulate the effect of the treatment, including both the intervention and biomarker values in the model could introduce multicollinearity issues, making the model more complicated without providing additional predictive value. Therefore, we believe that our current approach effectively captures the necessary information regarding treatment impact through the biomarkers themselves.

4. While the decay of performance post initial triage is unsurprising, repeating the PIERS model may still have value (albeit more modest) as suggested by NICE in identifying women at risk. This would mean undertaking a prospective study comparing a formal PIERS reassessment to current 'standard of care'- repeated bedside assessment. Do you have any information on this sort of comparison?

Thank you for your question. We have taken the fullPIERS model used repeatedly as the current standard of care in accordance with the NICE guidelines. For our assessment of performance over time, we needed a numerical estimate of risk, and we were not aware of an alternative to produce that other than the methods recommended in the guidelines.

The methodology is very comprehensive, and the graphs are sophisticated. As one not used to interpreting these novel visual representations, I did find them- and the accompanying text- very hard to digest. Others in the general readership may also find this difficult. This may benefit from examples or detailed explanations in the supps to improve readability and accessibility of the paper.

Thank you very much for your valuable suggestion*.* It is indeed a good idea. We have added a supplementary explanation to the complex figures. More specifically, we explained precision recall curve, decision curve analysis and Sankey diagram in details, and provided an example of precision recall curve.

Specific questions:
1) Given the miniPIERS model was initially developed for LMIC, why did you not compare the performance of this model as well?

Thank you for raising this important point. We conducted this study in response to the UK NICE guideline, which recommends the use of the externally-validated fullPIERS model for consecutive predictions. In contrast, the miniPIERS model is neither recommended for this purpose nor has it been externally validated, so it was not the focus of our analysis. Our study also examined the performance of the externally validated PIERS-ML model over time as it outperforms the fullPIERS model.

Given that the fullPIERS model demonstrated superior initial performance compared with the miniPIERS model but still exhibited significant performance degradation over time, we infer that the miniPIERS model would probably perform even less effectively under similar conditions.

2) The cohorts should be better described in terms of years of recruitment, gestational age etc. Were these patients all preterm undergoing expectant management (line 101)? Information about GA helps to interpret the rapid fall in number of patients providing measurements across time.

Thank you very much for this insightful suggestion. We have revised our manuscript accordingly. Table S1 is added that shows the baseline characteristics of the women. The gestational age of the women at admission ranges from 30.57 to 38.63 and the women were recruited between year 2003 and 2016 (see lines 94 – 95 in the clean copy). Please note our response to the question of expectant management, above.

3) is there a reason for not using the iHOPE core outcome set which includes perinatal outcomes? I presume this is because you didn't have the variables collected given some of these cohorts dated back to 2003. Please clarify.

Thank you for raising this important question. The combined PIERS outcome of this study was developed by an iterative Delphi consensus prior to the development of the iHOPE core outcome set (to which the PIERS investigators contributed). The two lists are very close in content, but there are important differences. For example, HELLP syndrome is included as an element of the iHOPE core outcome set. In our view, remote from term, careful practitioners will await the development of HELLP syndrome to guide shared decision making about expedited delivery, to avoid the hepatorenal adverse outcomes that are in the PIERS combined outcome. Therefore the label of ‘HELLP’ is not an outcome that practitioners wish to avoid, but rather the most severe consequences of HELLP. This approach has been supported by our PPIE advisors, the Preeclampsia Foundation, throughout this journey (since 2001).

We believe that there is a competing risk between the mother and the fetus, and that it may be more useful to have a clear estimate of the maternal risk, and assess the fetal risk separately with the standard clinical methods (most NICUs know their own outcome rates for a given gestational age at delivery, birthweight, and sex) or specific fetal outcome models.

4) Can you please explain why you think the fullPIERS model performed so poorly at baseline (Fig S2) compared to ML PIERS (Fig 1)

This may have resulted from the following aspects: (1) The PIERS-ML model was trained on a significantly larger dataset compared to the fullPIERS model, which was developed over a decade ago; (2) The PIERS-ML model utilized a machine-learning algorithm capable of capturing complex interactions among predictors, potentially enhancing predictive accuracy, especially with a sufficiently large sample size; (3) The baseline performance of the PIERS-ML model was assessed on its development dataset, which can lead to overestimated performance (it is important to note that the PIERS-ML model has also been externally validated on a different dataset). In contrast, the fullPIERS model was evaluated on an external validation dataset, where lower performance is more expected. Recalibrating the fullPIERS model may improve its performance when applied in different countries.[4]

5) This paper assumes quite a lot of knowledge, particularly from the Lancet Digital Health paper. I would suggest at least providing an example of a Precision Recall plot so the readership can understand how the summary AUCs have been generated that are subsequently presented in Fig 2 and Fig S3. Also it would help if the y axis was identical in these 2 graphs so the different performance of full PIERS and ML PIERS was immediately visible.

Thank you very much for the very helpful suggestions. We have provided an example of a Precision Recall plot in the supplementary explanation to complex figures that explains in detail how the summary AUCs have been generated. And we have we have updated Fig 2 and Fig S3 to make sure they have identical y axis for easier comparison of the results.

6) The decision curve analyses are quite complex to follow.  Further, it's said that the model is better than treat all for prob of 3.1% on Day 0, but this isn't clearly apparent in the figure (referenced Fig 3b, but should be fig 3a, I think). Treat all is better for this group on Day 4 (Fig 3b). The statement that these 'align with previous findings' is not referenced (line 317).

Thank you very much and for the efforts in reading our paper in detail. Indeed “treat all” is better for this group on Day 4 (Fig 3b). There was a typo in the sentence “*When the threshold probability was 3.1%, treatment guided by the model only yielded higher benefit (than treating all patients) on day 0 (Fig 3b)*”. “day 0” should be “day 4” which corresponds to Fig 3b. We have fixed this issue.

We apologize for the confusion in the statement that 'these align with previous findings'. By “previous findings” we meant the results presented earlier in this study. We have rephrased the text as follows (lines 348-349 in the clean copy): “… *aligned with the findings of other analyses in this study*.”

**Comments from Reviewer 3**: "Performance drift detected from consecutive prediction of adverse maternal outcomes of pre-eclampsia, using the PIERS-ML and fullPIERS models: A multi-country prospective observational study" examines the existing Pre-eclampsia Integrated Estimate of RiSk (PIERS) models for ongoing assessment beyond 48 hours, as the PIERS models had originally only been developed and validated as "static" models as part of an initial assessment within 48 hours. The multi-country prospective observational study assessed both PIERS-ML and fullPIERS models for each day within a two-week post-admission window, and found that while PIERS-ML had acceptable performance for specific risk groups, this was not the case for other risk groups beyond 48 hours. As such, it was recommended to use the PIERS models only for the first day, as originally intended and validated.

1. In the Data Source subsection, it is stated that "The baseline data of this pooled database... was used for development of the PIERS-ML model".  From [7], PIERS-ML was developed for outcomes within 2 days (and 7 days), using clinical data at admission (baseline data)

From what could be understood, "consecutive/ongoing prediction" would then be applying the PIERS-ML model developed only on the baseline data, to follow-up measurements (i.e. for say the 7th day post-admission, treat the clinical values from the 7th day as the baseline, to predict up to the 9th day for the PIERS-ML 2-day model). If this is correct, the perhaps obvious approach would seem to be to develop (and validate) separate PIERS-ML models for consecutive/ongoing prediction with the follow-up data (i.e. for each day as baseline); it is then curious as to why this does not appear to have been considered/discussed.

Thank you very much for your comments and the correct reflection on our paper. We conducted this study because the UK NICE guideline recommends using the fullPIERS model for consecutive predictions, but this model has not yet been validated for such application (see lines 81- 87 in the clean copy). We also analyzed the PIERS-ML model since it is an updated version of the fullPIERS model. We believe it is crucial to inform users about the performance of these models when used serially. If the models do not perform well in later stages, it may necessitate updating them or developing and validating new models, which would require a separate study.

The approach you suggested is indeed a valid method for solving the problem of performance drift. This approach would entail building new models based on existing models, which includes recalibrating intercept or both intercept and joint effects of predictors on the new follow-up measurements etc., as we have described in lines 373 to 376 in the clean copy: “*For consecutive prediction using serial measurements, existing prediction models can be updated or new models trained for this purpose. Potential approaches include recalibrating intercept or both intercept and joint effects of predictors*…”. However, this approach would result in multiple models and does not account for the trajectory of the predictors which might enhance predictive accuracy. Therefore, we are currently working on a dynamic version of the PIERS-ML model. We aim to have one model that incorporates predictor dynamics.

2. While performance drift was detected, this does not seem very surprising as-is, since one might expect outcomes of pre-eclampsia to vary (significantly) over time from admission. As such, while the analysis on existing static models may be instructive, the practical contribution of the study is less clear.

Thank you very much for your insightful comment. We agree that performance drift might be expected, given that the models were trained using only baseline data but were applied to subsequent days. However, it is important to note that without solid analysis, it is difficult to conclude definitively that the models perform increasingly worse over time.

Moreover, much as "performance drift" might be inferred qualitatively (with uncertainty), our study adds significant value by providing a quantitative assessment of this drift. Our results offer clinical audiences a clearer understanding that, although the models can be applied beyond the day of admission, their performance becomes increasingly unreliable within just a few days. This highlights the need to interpret model outputs more cautiously. Moreover, our results establish a benchmark which future new prediction models can be compared with to assess whether they offer improvements over static models.

3. In the Strengths and Limitations section, a limitation was stated as PIERS-ML and fullPIERS being partly evaluated on baseline data used for development. This appears to be a fairly critical concern pertaining to accurate evaluation, and a more convincing evaluation would be to perform the analysis only on test data not involved in the development of PIERS-ML/fullPIERS, as well as additional external evaluation data.

We totally understand your concern. Thank you very much for bringing it up. We would like to clarify that no data beyond day 0 was used for developing either the fullPIERS or PIERS-ML model. The present study showed the performance of the two models used for consecutive prediction **on the follow-up measurements** on the largest available dataset in this field. So actually we did the analysis as you suggested. The performance of both models on day 0 was only shown as we assumed readers would like to know, and it seems more fair to show the performance on day 0 using the same cohort.

The limitation we stated is only relevant to the performance of the PIERS-ML model on day 0 as the day 0 data were used for developing the model. The PIERS-ML model has been externally validated (AUROC: 0·80 [95% CI 0·76–0·84])[5] ,but we don’t have follow-up measurements in the external validation dataset. As for the fullPIERs model (the target model recommended to be employed for consecutive prediction in the NICE guideline), which was **built on data of 2023 women**,[1] we **evaluated its performance in this study on 8843 women including multiple external validation cohorts of this model**.

In conclusion, we aimed to signal the problem of performance drift, instead of validating newly-developed models. We interpret as strong evidence the fact that we detected performance drift even on the most optimistic cohort.

References

1.	von Dadelszen P, Payne B, Li J, Ansermino JM, Pipkin FB, Côté A-M, et al. Prediction of adverse maternal outcomes in pre-eclampsia: development and validation of the fullPIERS model. The Lancet. 2011;377(9761):219-27. doi: 10.1016/s0140-6736(10)61351-7.

2.	Payne B, Hodgson S, Hutcheon JA, Joseph KS, Li J, Lee T, et al. Performance of the fullPIERS model in predicting adverse maternal outcomes in pre-eclampsia using patient data from the PIERS (Pre-eclampsia Integrated Estimate of RiSk) cohort, collected on admission. BJOG. 2013;120(1):113-8. Epub 20121019. doi: 10.1111/j.1471-0528.2012.03496.x. PubMed PMID: 23078362.

3.	Ukah UV, Payne B, Karjalainen H, Kortelainen E, Seed PT, Conti-Ramsden FI, et al. Temporal and external validation of the fullPIERS model for the prediction of adverse maternal outcomes in women with pre-eclampsia. Pregnancy Hypertens. 2019;15:42-50. Epub 20180105. doi: 10.1016/j.preghy.2018.01.004. PubMed PMID: 30825926.

4.	Ukah UV, Payne B, Lee T, Magee LA, von Dadelszen P, fullPiers, mini PWG. External Validation of the fullPIERS Model for Predicting Adverse Maternal Outcomes in Pregnancy Hypertension in Low- and Middle-Income Countries. Hypertension. 2017;69(4):705-11. Epub 20170206. doi: 10.1161/HYPERTENSIONAHA.116.08706. PubMed PMID: 28167685.

5.	Montgomery-Csoban T, Kavanagh K, Murray P, Robertson C, Barry SJE, Vivian Ukah U, et al. Machine learning-enabled maternal risk assessment for women with pre-eclampsia (the PIERS-ML model): a modelling study. Lancet Digit Health. 2024;6(4):e238-e50. doi: 10.1016/S2589-7500(23)00267-4. PubMed PMID: 38519152; PubMed Central PMCID: PMCPMC10983826.

6.	National Institute for Health and Care Excellence: Guidelines. Hypertension in pregnancy: diagnosis and management. London: National Institute for Health and Care Excellence (NICE)

Copyright © NICE 2019.; 2019.

7.	Millman AL, Payne B, Qu Z, Joanne Douglas M, Hutcheon JA, Lee T, et al. Oxygen saturation as a predictor of adverse maternal outcomes in women with preeclampsia. J Obstet Gynaecol Can. 2011;33(7):705-14. doi: 10.1016/S1701-2163(16)34955-6. PubMed PMID: 21749746.
